# Visceromotor roots of aesthetic evaluation of pain in art: an fMRI study

**DOI:** 10.1093/scan/nsab066

**Published:** 2021-05-14

**Authors:** Martina Ardizzi, Francesca Ferroni, Maria Alessandra Umiltà, Chiara Pinardi, Antonino Errante, Francesca Ferri, Elisabetta Fadda, Vittorio Gallese

**Affiliations:** Department of Medicine and Surgery, University of Parma, 43126, Parma, Italy; Neuroscience & Humanities Lab, University of Parma, 43125, Parma, Italy; Department of Medicine and Surgery, University of Parma, 43126, Parma, Italy; Neuroscience & Humanities Lab, University of Parma, 43125, Parma, Italy; Neuroscience & Humanities Lab, University of Parma, 43125, Parma, Italy; Department of Food and Drug, University of Parma, 43124, Parma, Italy; Department of Art History Columbia University, Italian Academy for Advanced Studies, Columbia University, 10027, New York, NY, USA; Department of Neuroradiology, Fondazione IRCCS Istituto Neurologico Carlo Besta, 20133, Milan, Italy; Department of Medicine and Surgery, University of Parma, 43126, Parma, Italy; Department of Neuroscience, Imaging and Clinical Science, University G. d’Annunzio, 66100, Chieti, Italy; Neuroscience & Humanities Lab, University of Parma, 43125, Parma, Italy; Department of Humanities, Social Sciences and Cultural Industries, University of Parma, 43125, Parma, Italy; Department of Medicine and Surgery, University of Parma, 43126, Parma, Italy; Neuroscience & Humanities Lab, University of Parma, 43125, Parma, Italy; Department of Art History Columbia University, Italian Academy for Advanced Studies, Columbia University, 10027, New York, NY, USA

**Keywords:** art, anterior insula, cingulate cortex, inferior frontal gyrus, pain

## Abstract

Empathy for pain involves sensory and visceromotor brain regions relevant also in the first-person pain experience. Focusing on brain activations associated with vicarious experiences of pain triggered by artistic or non-artistic images, the present study aims to investigate common and distinct brain activation patterns associated with these two vicarious experiences of pain and to assess whether empathy for pain brain regions contributes to the formation of an aesthetic judgement (AJ) in non-art expert observers. Artistic and non-artistic facial expressions (painful and neutral) were shown to participants inside the scanner and then aesthetically rated in a subsequent behavioural session. Results showed that empathy for pain brain regions (i.e. bilateral insular cortex, posterior sector of the anterior cingulate cortex and the anterior portion of the middle cingulate cortex) and bilateral inferior frontal gyrus are commonly activated by artistic and non-artistic painful facial expressions. For the artistic representation of pain, the activity recorded in these regions directly correlated with participants’ AJ. Results also showed the distinct activation of a large cluster located in the posterior cingulate cortex/precuneus for non-artistic stimuli. This study suggests that non-beauty-specific mechanisms such as empathy for pain are crucial components of the aesthetic experience of artworks.

## Introduction

Neuroimaging studies demonstrate that the observation of other individuals’ somatosensory experiences or emotional facial expressions recruits sensory, premotor and visceromotor brain areas involved also in the first-person experience of the same state (Carr *et al.*, 2003; Wicker *et al.*, 2003; Ebisch *et al.*, 2008, 2011; Caruana *et al.*, 2017). Our hypothesis is that this common neural ground for experiencing and observing emotions also serves the formation of an aesthetic judgement (AJ) when the emotional content of the artistic work is represented at a figurative level. To address our hypothesis, we focus on empathy for pain triggered by artistic and non-artistic painful facial expressions. Among the numerous cerebral regions active both during the first-person experience of pain and the mere observation of others’ facial expressions of pain, the bilateral anterior insula (AI)/fronto-insular cortices and the cingulate cortex (CC)—especially in its anterior medial (aMCC) and posterior anterior (pACC) sectors—are, indeed, consistently identified (Botvinick *et al.*, 2005; Lamm *et al.*, 2011). This functional overlap suggests that empathy for pain is underpinned by visceromotor and viscerosensitive neural structures that are also involved in the direct experience of pain. Indeed, the insular cortex is characterized by a clear anatomical and functional caudal-to-rostral gradient of increasingly complex integration of bodily signals. Whereas the direct experience of pain is somatotopically mapped only in the posterior insular subdivision associated with the sensory components of nociception (Ostrowsky *et al.*, 2002; Kurth *et al.*, 2010), visceromotor and viscerosensitive responses are related to the activation of the AI, induced both during the actual experience of pain and by the observation of others’ pain facial expressions (Ostrowsky *et al.*, 2000; Lamm *et al.*, 2011; Benuzzi *et al.*, 2018). Interestingly, although far from being a pain-specific region (Kurth *et al.*, 2010), when the AI is damaged by brain lesions, patients worsen their recognition of another person’s pain experience, suggesting its causal involvement in other’s pain detection (Gu *et al.*, 2012). At the same time, mounting evidence supports the role of the pACC and aMCC in autonomic and motor control. A recent study (Caruana *et al.*, 2018) demonstrates that the intracerebral high-frequency electrical stimulation of the aMCC in a large cohort of drug-resistant epileptic patients triggers a variety of goal-oriented and defensive behaviours involving the upper limbs or the entire body. Differently, the adjacent pACC appears to be involved in the production of facial emotional displays and autonomic responses identified by the patients as fear and anxiety (Caruana *et al.*, 2018). Coherently, a study conducted on rats’ homologous mesial regions found neurons responding both when rats experience pain, as triggered by a laser, and while they witness another rat receiving shocks (Carrillo *et al.*, 2019). These authors also demonstrate that the deactivation of this region reduces rats’ nocifensive behaviours (i.e. freezing) while observing a conspecific experiencing painful shocks.

The existence of a common neural ground for experiencing and observing emotions has been mainly investigated as the functional mechanism underpinning the recognition of others’ emotions (Gallese *et al.*, 2004; Gallese and Sinigaglia, 2011; Gallese, 2014). However, this functional mechanism has been proposed to also play a role in other contexts. For example, when reading single words with threat connotation, not only the visuo-linguistic cerebral nodes but also the amygdala, a key region for the direct experience of fear, are recruited (Weisholtz *et al.*, 2015). In a similar vein, these vicarious functional brain activations have been linked to aesthetic experience (Freedberg and Gallese, 2007; Gallese, 2017).

Aesthetic experience has been defined as a complex and specific emergent mental state arising from the interaction of emotion–evaluation, sensorimotor and meaning–knowledge processes (Chatterjee and Vartanian, 2016). Both bottom-up and top-down processes concur to the specificity of the neurocognitive underpinnings of the aesthetic appreciation of art (Pearce *et al.*, 2016; Pelowski *et al.*, 2017). In this field, seminal functional studies investigated the neural correlates of aesthetic experience, trying to localize cerebral regions sensible to beauty. The faculty of beauty seems to recruit the medial orbitofrontal (Ishizu and Zeki, 2011; Ishizu, 2014; Zeki *et al.*, 2014) and the dorsolateral prefrontal cortices (Cela-Conde *et al.*, 2004; Cattaneo *et al.*, 2014). This brain-based approach to the study of aesthetics suggests that all works of art that appear beautiful to a subject affect the activity of specific brain regions (Ishizu and Zeki, 2011). Another approach is to investigate whether neural circuits that we know are linked to functions not associated with the experience of beauty (e.g. empathy for pain brain regions) can also play a role in the formation of aesthetic experience. Among these non-beauty-specific neural mechanisms possibly underpinning the aesthetic power of images, convergent studies propose the observer’s sensory and visceromotor engagement with images as a valid candidate. Behavioural studies show that observer’s simulation of artists’ creative gestures increases the aesthetic evaluation of paintings made with congruent hand movements (Leder *et al.*, 2012; Ticini *et al.*, 2014; McLean *et al.*, 2015). Direct demonstration of the link between activation in premotor cortices and AJ can be found in studies investigating the neural correlates of dance enjoyment (for a review, see Kirsch *et al.*, 2016). In a pioneering study, Calvo-Merino and colleagues (2008) found a significant activation in the premotor cortex during passive viewing of dance stimuli that was related to the subsequent aesthetic evaluation of the same stimuli. More recently, using stimuli depicting static or dynamic representational paintings of human figures or landscapes, a link—mediated by dynamism impression—between the amplitude of observers’ motor evoked potentials and their liking judgements has been demonstrated (Fiori *et al.*, 2020). Coherently, the AJ of landscape paintings involves the posterior and central sectors of the insular cortex, in relation to the intrinsic dynamism of the artwork (Di Dio *et al.*, 2016). Also, observers’ sensorimotor engagement with portrayals of painful facial expressions influences their explicit AJs (Ardizzi *et al.*, 2020a). Specifically, it has been found that the overt contraction of the corrugator supercilii facial muscle increased the aesthetic rating of artistic facial expressions of pain, where the contraction of the same facial muscle was visible. This latter result seems to support Schott’s claim about the potential engagement of empathy for pain brain regions (i.e. sensory, premotor and visceromotor brain areas) during the aesthetic appreciation of pictorial representation of pain (Schott, 2015). Until now, no studies have directly explored this hypothesis. Only two neuroimaging studies not specifically interested in aesthetic appreciation but using pictorial representation of injured bodies (De Gelder *et al.*, 2018) or mourning scenes (Labek *et al.*, 2017) offered mixed results in support of the recruitment of empathy for pain brain regions during the enjoyment of such artistic stimuli.

In the present functional magnetic resonance imaging (fMRI) study, we investigate the brain activations related to the vicarious pain experience triggered by artistic and non-artistic stimuli with the following aims: (i) to elucidate whether the observation of artistic facial expressions of pain is able to activate the brain regions normally associated with empathy for pain; (ii) to understand whether this specific activation pattern could be involved in the AJ of the same images and (iii) to verify whether the two vicarious experiences of pain, one induced by art and the other aroused by non-artistic stimuli, evoke different brain activation patterns.

## Materials and methods

### Participants

Twenty healthy right-handed volunteers with no training in art or art history [11 females; mean age = 25.15, Standard Error (SE) = 0.68, mean schooling = 15.25, SE = 0.41; mean Art Experience Questionnaire (Chatterjee *et al.*, 2010) score = 12.5, SE 1.89] participated in the study. Handedness was assessed by means of the Edinburgh Inventory (Oldfield, 1971). All participants had normal or corrected-to-normal visual acuity. No participant had a history of neurologic, general medical or psychiatric conditions. The experimental protocol was approved by the Ethics Committee of the University of Parma, and it was in line with the Declaration of Helsinki 2013. Written informed consents were collected from all participants.

### Stimuli

Twenty-four high-resolution digital versions of neutral (*N = *12) and painful (*N = *12) facial expressions were used as experimental stimuli. Half of the stimuli were selected from Renaissance and Baroque paintings [Art Pain (AP) stimuli, *N = *6; Art Neutral (AN) stimuli, *N = *6], whereas the other half derived from non-artistic digital photographs of models’ facial expressions [non-Art Pain (nAP) stimuli, *N = *6; non-Art Neutral (nAN) stimuli, *N = *6]. Stimuli selection followed recent guidelines for the use of artworks as stimuli in empirical research (Hayn-Leichsenring, 2017). Please see Supplementary Material for a detailed description of the procedure followed to select images and validate the final set of stimuli.

### Experimental design

**Fig. 1.**
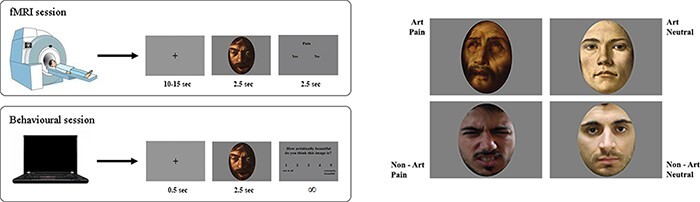
Experimental design and stimuli. In the fMRI session, participants judged if the face depicted in the stimulus showed an expression of pain or not. In the behavioural session, performed outside the scanner, participants were asked to express an AJ on a 5-point ordinal scale. Four exemplificative stimuli are displayed in the right panel of the figure.

The experimental protocol consisted of two sessions (see Figure 1):

#### fMRI session.

Participants lay in the scanner in a dimly lit environment. The stimuli were viewed via digital visors (VisuaSTIM) with a 500 000 pixel × 0.25 square inch resolution and horizontal eye field of 30°. The digital transmission of the signal to the scanner occurred via optic fibre. The software E-Prime 2 Professional (Psychology Software Tools, Inc., Pittsburgh, USA, http://www.pstnet.com) was used both for stimuli presentation and the recording of participants’ answers.

Participants were instructed to indicate, on the appearance of the task question, if the face depicted showed an expression of pain or not. Responses were given using the index or medium fingers of their right hands. Options’ order was balanced across participants congruently with the screen that appeared in the scanner (i.e. ‘Pain: Yes or No’; ‘Pain: No or Yes’).

The experiment consisted of six runs lasting 7 minutes each. The total duration of the entire experiment was approximately 50 minutes. Each run consisted of 24 randomized trials, six for each condition (i.e. AP, AN, nAP and nAN), constituting the event-related fMRI design. Each stimulus was presented six times across the six runs. Each trial began with a central fixation cross (ranging from 10 to 15 sec; i.e. implicit baseline of the subsequent functional analyses) followed by stimuli presentation lasting 2.5 sec. After stimulus presentation, the task question (i.e. ‘Pain: Yes or No’ or ‘Pain: No or Yes’) lasting 2.5 sec appeared. Overall, the experiment consisted of 144 trials, 36 for each condition. Before the beginning of the fMRI session, an out-of-scanner training of eight stimuli (two for each condition), different from those showed in the following scan session, was administered to ensure that participants understood the instructions and became familiar with timing and also with the use of the dial.

#### Behavioural session.

Immediately after the fMRI session, the stimuli were shown again in the AJ task administered outside the scanner. Participants were asked to answer the question ‘How artistically beautiful do you think this image is?’ using a 5-point ordinal scale ranging from ‘not at all’ (1) to ‘extremely beautiful’ (5). AJ task consisted of 144 randomized trials, 36 for each condition. Each trial began with a central fixation cross lasting 0.5 sec followed by stimulus presentation lasting 2.5 sec. After this period, task question and ordinal scale appeared. The next trial began after participants’ no-time-limit answers. The entire duration of AJ task was approximately 12 minutes, depending on participants’ response time. Lastly, participants were required to respond in a yes/no forced choice task whether they had seen the stimuli before the study. All participants reported that they had not seen any of the images before (100% unfamiliarity rating).

### fMRI data acquisition

Anatomical T1-weighted and functional T2*-weighted MR images were acquired with a 3-Tesla General Electric scanner equipped with an 8-channel receiver head coil. Functional images were acquired using a T2*-weighted gradient-echo, echo-planar (EPI) pulse sequence (acceleration factor asset = 2, 40 sequential transverse slices covering the whole brain, with a Repetition Time (TR) time of 2.5 sec, Echo Time (TE) = 30 msec, flip angle = 90°, Field of View (FOV) = 205 × 205 mm^2^, inter-slice gap = 0.5 mm, slice thickness = 3 mm, in-plane resolution = 2.5 × 2.5 × 2.5 mm^3^). At the end of the six functional runs, a T1-weighted anatomical scan (acceleration factor arc = 2, 156 sagittal slices, matrix 256 × 256, isotropic resolution 1 × 1 × 1 mm^3^, Time to Invert (TI) = 450 msec, TR = 8100 msec, TE = 3.2 msec, flip angle = 12°) was acquired for each participant.

### Statistical analyses

Data analysis was performed with SPM12 (Statistical Parametric Mapping software; The Wellcome Department of Imaging Neuroscience, London, UK; http://www.fil.ion.ucl.ac.uk) running on MATLAB R2017b (The Mathworks, Inc., Natick, MA) and IBM SPSS statistics 24. The first four volumes of each run were discarded to allow for T1 equilibration effects. For each participant, all volumes were corrected for slice timing using the middle slice as reference. Then, all volumes were spatially realigned to the first one of the first session and un-warped to correct for between-scan motion, and a mean image from the realigned volumes was created. T1-weighted images was co-registered to the mean fMRI volume; then segmented into grey, white and cerebrospinal fluid; and finally spatially normalized to the Montreal Neurological Institute (MNI) coordinates system. The thereby derived spatial transformation by co-registered T1 normalization was applied to the realigned EPI volumes, which after normalization were re-sampled in 1 × 1 × 1 mm^3^ voxels using trilinear interpolation in space. All functional volumes were then spatially smoothed with a 6 mm full-width half-maximum isotropic Gaussian kernel for the group analysis.

Data were analysed using a random-effects model (Friston *et al.*, 1999), implemented in a two-level procedure. In the first level, single-subject fMRI responses were modelled in a General Linear Model by a design matrix comprising the onsets and durations of each event for each functional run (AP, AN, nAP, nAN and Response). Trials erroneously identified by participants were regressed separately. No participant exceeded the 16% of incorrect trail identification. This analysis employed event-related convolution models using the hemodynamic response function provided by SPM12. The presentation of the stimuli for each trial condition was modelled as mini-epoch lasting 2.5 sec, whereas the motor response was modelled as one punctual single event. Motion regressors were included to control for possible artefacts related to head motion. For all participants, head motion never exceeded 3 mm.

In the second-level analysis (group analysis), corresponding contrast images from the first level for each participant were entered into flexible analysis of variance (ANOVA) with sphericity correction for repeated measures (Friston *et al.*, 2002). This model considered the patterns of activation obtained for the main effects—Emotion (Pain, Neutral) and Content (Art, Non-Art), as well as the interaction between the two factors (Emotion × Content). All results were thresholded at *P* < 0.05 family wise error (FWE) corrected at the cluster level (cluster size estimated with a voxel-level threshold of *P*-uncorrected = 0.001).

To highlight voxels activated both by painful facial expression whether artistic or non-artistic, a conjunction analysis was performed between AP *vs* implicit baseline and nAP *vs* implicit baseline, adding an inclusive mask derived from the main effect of Emotion (mask thresholded at *P* < 0.05 FWE corrected at cluster level). The mask inclusion limited the results of the conjunction analysis to brain regions involved in emotional processing. To appreciate the whole-brain functional activations associated with each condition of the present study, statistical maps were also obtained contrasting each stimulus category (AP, AN, nAP and nAN) *vs* implicit baseline (i.e. fixation cross) (see Supplementary Material).

The location of the activation foci was determined in the stereotaxic space of the MNI coordinates system using the probabilistic maps of the human brain included in SPM Anatomy Toolbox v1.7 (Eickhoff *et al.*, 2005).

Three regions of interest (ROIs) were created, based on a previous meta-analysis study (Lamm *et al.*, 2011), on the left and right AI (lAI and rAI) and on the CC using MarsBaR Toolbox for SPM (release 0.44). All ROIs were defined centring the sphere (radium = 10 mm) around the maxima of these clusters (ROI rAI: *x* = 39, *y* = 23, *z* = −4; ROI lAI: *x* = −40, *y* = 22, *z* = 0; ROI CC: *x* = −2, *y* = 23, *z* = 40). These regions were reported as consistently activated across the 32 studies included both in the coordinate- and image-based meta-analysis (Lamm *et al.*, 2011). Furthermore, the selected ROIs are also close to the activation foci associated with the main effect of Emotion in the present study. Mean beta weights associated with the contrast images, AP *vs* AN, of each participant were extracted using REX Toolbox (Duff *et al.*, 2007). Coherent with this investigated contrast, ROIs’ mean beta weights were correlated with the change score between the mean AJ attributed to AP and the same assigned to AN (ΔAJ_A_= AJ_AP_ − AJ_AN_). Three Pearson’s two-tailed correlation analyses were then performed between ROIs mean beta weights and ΔAJ_A_.

The specificity of this effect was investigated by performing an additional correlation analysis between ΔAJ_A_ and mean beta weights extracted from a control brain region not directly involved in other’s pain detection but related to face perceptual analysis, the Fusiform Face Area (FFA) (ROI FFA: *x* = 42, *y* = −50, *z* = −19; Cohen *et al.*, 2019). One participant was removed from ROIs analyses due to technical problems in the recording of the responses in the behavioural session. Consequently, ROIs analyses were performed on 19 participants.

See Supplementary Material for the results of the AJ task.

## Results

In the fMRI session, brain activity was measured as a function of Emotion (Pain, Neutral), Content (Art, Non-Art) and their interaction (Emotion × Content). fMRI results are listed in Table 1.

**Table 1.** Main effects and interaction from functional ANOVA analysis

						Local maxima (MNI)		
	Brain structure	Side	Cluster extent size in voxel (Ke)	*P* FWE corrected at the cluster level	*Z*	*x*	*y*	*z*
Main effect of Emotion	pACC/aMCC/MFG	R/L	9623	<0.001	5.58	−1	28	39
					4.52	4	36	28
					4.04	9	20	56
	AI/IFG	R	8003	<0.001	5.10	44	22	−6
					4.55	39	11	45
					4.37	54	30	25
	AI/IFG	L	5061	<0.001	5	−42	22	−3
					4.12	−47	13	8
Main effect of Content	PCC/precuneus	R/L	1086	0.027	4.51	3	−60	28
					3.51	−6	−58	25
Emotion × Content	IOG	R	1357	0.009	5.36	31	−93	−5
					3.26	35	−92	−12

Results are thresholded at *P* < 0.05 FWE corrected at the cluster level. Local maxima are given in MNI standard brain coordinates. Most probable anatomical regions are derived from Anatomy Toolbox 1.7 (Eickhoff *et al.*, 2005) and listed in ‘Brain structure’ column.

**Fig. 2.**
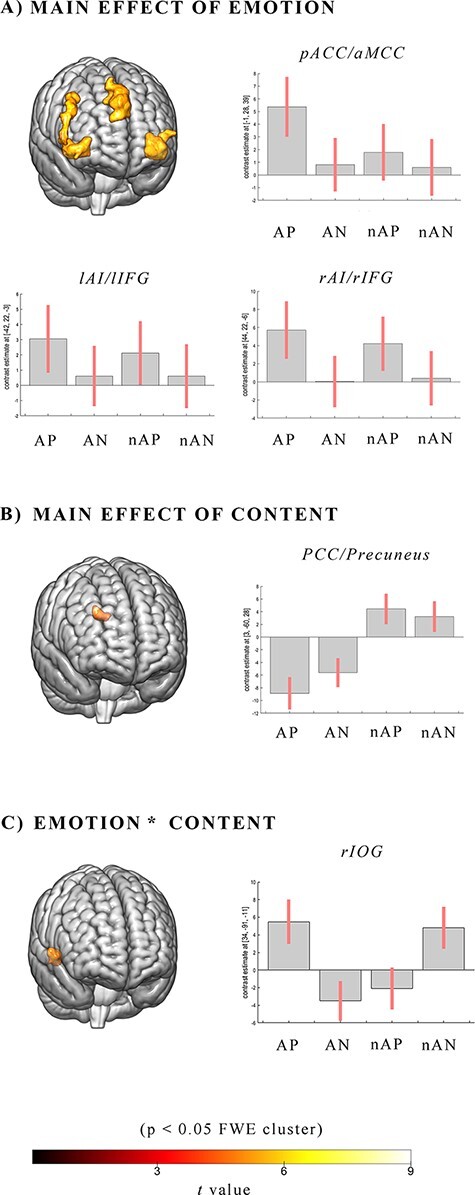
Brain activation maps observed for the main effect of Emotion (A), Content (B) and their interaction (C). The plots show the activity profile for AP and AN stimuli, nAP and nAN stimuli in arbitrary units, +/2 10% confidence intervals (*P* < 0.05_FWE corrected at the__cluster level_). Group-average statistical parametric maps are rendered onto the MNI brain template (*P* < 0.05_FWE corrected at__the__cluster level_).

The main effect of Emotion revealed a quite extensive activation for painful facial expressions, irrespective of Content, in the pACC and aMCC partially extending to the medial frontal gyrus (MFG). Additional activations were present bilaterally in large clusters including the AI and inferior frontal gyrus (IFG); the latter is more extended into the right hemisphere (Figure 2, panel A).

The main effect of Content revealed significant activation of the posterior CC (PCC)/precuneus mainly evoked by non-artistic stimuli (Figure 2, panel B).

The interaction Emotion × Content showed increased activation in a cluster located in the right inferior occipital gyrus (rIOG) extending to the calcarine gyrus, with AP and nAN producing greater activations than AN and nAP (Figure 2, panel C).

**Fig. 3.**
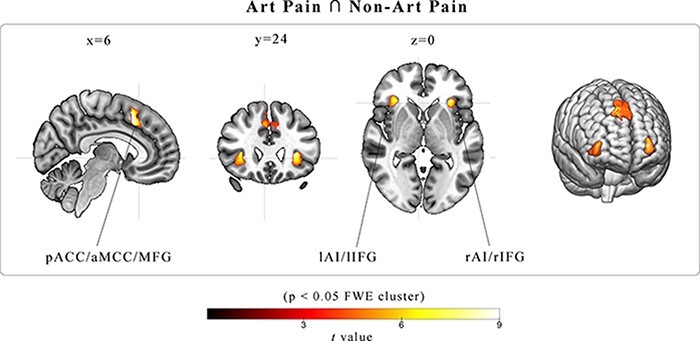
Brain activation map resulting from the conjunction analysis. The map is obtained from the conjunction between the contrasts AP *vs* baseline and nAP *vs* baseline, masked using an inclusive contrast image derived from the main effect of Emotion. Group-averaged statistical parametric maps are rendered into a standard MNI brain template and in three representative slices (*P* < 0.05_FWE corrected at__the__cluster level_).

The results of the conjunction analysis (AP ∩ nAP; Figure 3; Table 2) showed that both in AP and nAP conditions there were significant activations in a midline cluster encompassing the pACC, aMCC and MFG, as well as, in two bilateral clusters including the AI and the IFG.

**Table 2.** Results of conjunction analysis

						Local maxima (MNI)		
Contrast	Brain structure	Side	Cluster extent size in voxel (Ke)	*P* FWE corrected at the cluster level	*Z*	*x*	*y*	*z*
AP ∩ nAP	pACC/aMCC/MFG	R/L	2569	0.001	7.31	−5	13	47
					6.99	7	16	43
					6.49	7	15	52
	AI/IFG	L	1298	0.022	6.35	−29	27	3
					3.26	−37	16	2
	AI/IFG	R	1536	0.01	5.83	31	27	5

Results are thresholded at *P* < 0.05 FWE corrected at the cluster level. Local maxima are given in MNI standard brain coordinates. Most probable anatomical regions are derived from Anatomy Toolbox 1.7 (Eickhoff *et al.*, 2005) and listed in ‘Brain structure’ column.

**Fig. 4.**
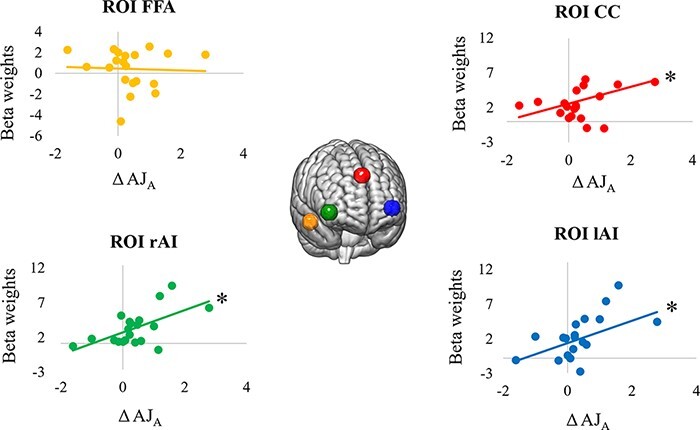
Pearson’s correlation analyses conducted between mean beta weights and AJs. Mean beta weights were extracted from left (in blue) and right (in green) AI and from the CC (in red). As a control region, mean beta weights were obtained from the FFA (in orange). *P < 0.05.

Pearson’s correlation analyses performed between ΔAJ_A_ and the beta weights extracted from the cingulum and the insular cortices were significant (ROI rAI: *r*_19_ = 0.7, *P* = 0.001; ROI lAI: *r*_19_ = 0.6, *P* = 0.006; ROI CC: *r*_19_ = 0.48, *P* = 0.04) (Figure 4). Differently, Pearson’s correlation analysis performed between ΔAJ_A_ and the beta weights extracted from the Fusiform Face Area was not significant (control ROI FFA: *r*_19_ = −0.009, *P* = 0.97).

## Discussion

Empirical evidence has consistently demonstrated that the recognition of others’ facial expression of emotions and sensations, like pain, is underpinned by the activation of the brain regions that are active during the subjective experience of the same emotions and sensations. However, new evidence (Leder *et al.*, 2012; Ticini *et al.*, 2014; Kirsch *et al.*, 2016; Fiori *et al.*, 2020; Ardizzi *et al.*, 2020a) suggests that this basic access to others’ emotions may also feed other high-level processes, such as the aesthetic experience of works of art and the formation of AJs. In the present study, we better explored this hypothesis investigating the brain activations associated with vicarious experiences of pain triggered by artistic or non-artistic images depicting facial expressions of pain. First, our aim was to understand whether artistic pain representations engage observers’ empathy for pain brain regions. Second, we wanted to understand whether the possible recruitment of this common brain network for experiencing and observing pain helps the formation of the AJ of non-art expert observers. Third, we are totally aware that witnessing others’ pain looking at the picture of a real face or at the pictorial representation of the same facial expression are two different experiences. For this reason, we expected to also find distinctive brain activations for artistic and non-artistic facial expressions of pain.

Considering the first aim of the present study, activations derived from the main effect of Emotion showed the expected empathy for pain brain regions’ activations (i.e. bilateral AI and pACC and aMCC). Interestingly, the bilateral activations in the AI significantly extend to the IFG, especially in the right hemisphere. IFG plays an important role in the motor mirror-neuron mechanism that likely supports recognition and imitation of actions (Rizzolatti and Sinigaglia, 2016), but it is also specifically related to the observation/evaluation and execution of facial expressions of emotions (Carr *et al.*, 2003; Hennenlotter *et al.*, 2005; Van Der Gaag *et al.*, 2007; Jabbi and Keysers, 2008). Indeed, patients with localized damages limited to IFG are selectively compromised in the recognition of facial expressions of emotions, but not in second-order false belief attribution (Shamay-Tsoory *et al.*, 2009). A more recent meta-analysis investigating the specific brain areas subserving specific sub-processes of mindreading found convergent activations in the IFG when others’ internal states are inferred from their faces or eyes (Schurz *et al.,* 2014). The authors concluded that IFG supports a particular form of mindreading made possible by a ‘common coding’ mechanism for action and perception, that is, the mirror mechanism. Coherently, in the context of painful facial expressions, IFG is involved in the detection of the emotional components of pain (Budell *et al.*, 2010, 2015).

In agreement with the results of the main effect of Emotion, the conjunction analysis performed between the whole-brain activations for AP and nAP stimuli revealed the activation of pACC/aMCC, together with bilateral AI/IFG clusters. The activation patterns revealed by the present study demonstrate that the activation of empathy for pain brain regions (i.e. AI, pACC and aMCC) and IFG can also be elicited by both artistic and non-artistic representations of facial pain. Other studies demonstrated that the observation of actions depicted in figurative works of art (Battaglia *et al.*, 2011; Thakral *et al.*, 2012) stimulates the responses of sensorimotor circuits also involved in actual motor control. However, for the first time, the present results provide evidence that the common neural network for actual and vicarious emotional experiences can also be elicited by art emotional content. When observers try to decode others’ painful facial expressions, be they produced by an expressive natural behaviour or created by intentional artistic practice, both lead to the vicarious activation of a set of brain areas relevant to the direct experience of the same emotional state.

We moved a step forward in the attempt to meet our second aim and link this neural activation pattern to the formation of AJ. Results show that the activations found in empathy for pain brain regions are also positively correlated with participants’ AJs attributed to artistic facial expressions of pain with respect to artistic neutral faces. In other words, the higher the response of these areas, the higher the AJ of artistic beauty of painful facial expressions. As expected, this relation is present only for empathy for pain brain regions and not for brain areas involved in face perceptual analysis but not in others’ pain detection. These findings support the idea that the activation of empathy for pain brain circuit and of IFG is involved in the AJ of beauty when the source of the emotional content is artistic. Even in the context of contemporary dance enjoyment, the activations of IFG and other mentalizing areas were associated with spectators’ grasping of dance coherence (Bachrach *et al.,* 2016). Interestingly, Ishizu and Zeki (Ishizu and Zeki, 2017) found that when people aesthetically rate sorrowful works of art, the brain areas involved in the empathic experience of other people’s sadness are functionally connected to regions implicated in the judgement of beauty, suggesting how empathic engagement and aesthetic experience are two interrelated phenomena.

In a similar vein, the present results on the aesthetic involvement of visceromotor and premotor brain regions related to the experience of pain suggest that the empathic engagement with works of art concerns a bodily-based direct access to art emotional content. Indeed, motor and physiological responses coherent with the artistic emotional climax are widely demonstrated across many forms of art (Lundqvist *et al.*, 2009; Koelsch, 2014; Wassiliwizky *et al.*, 2017; Siri *et al.*, 2018; Kaltwasser *et al.*, 2019; Ardizzi *et al.*, 2020b). The absence of a significant correlation between FFA activation and AJ for artistic images outlines the specificity of such relation for empathy for pain brain regions and stimulates some considerations about the qualification of this functional mechanism in aesthetic appreciation. For example, Cattaneo and collaborators (Cattaneo *et al.*, 2015) interfering with extra-striate area V5 suppressed both the perceived sense of motion and the liking of abstract but not of representational paintings. As in Cattaneo *et al.* (2015), where the dynamism perceived in abstract paintings drove their aesthetic appreciation, our results show that the direct access to the observed pain experience concurs with the AJ of painful facial expressions as represented in paintings. Overall, these results suggest that non-beauty-specific sensory and visceromotor engagement with images can contribute, under specific circumstances, to the formation of AJ, linking together the emotion–evaluation, sensorimotor and meaning–knowledge processes composing the aesthetic experience (Chatterjee and Vartanian, 2016; Ardizzi, 2020).

As stated before, the decoding of artistic facial expression and the decoding of non-artistic facial expressions of pain are not two perfectly overlapping processes. The results of the main effect of Content showed the activation of a large cluster located in the PCC/precuneus for non-artistic stimuli. These regions turned out to be responsive to a wide range of highly integrated tasks, including visuospatial imagery, visual information processing, episodic memory retrieval and self-processing operations, such as first-person perspective taking and the experience of agency (Cavanna and Trimble, 2006). Thanks to these functional activations, it is not surprising that the precuneus was seen to be consistently activated in mentalizing tasks (Molenberghs *et al.*, 2016). The activation of these regions suggests the preferential recruitment of visual and self-referential processing when decoding others’ emotional states, as when portrayed by non-artistic facial expressions. The results of the interaction Emotion × Content showed interesting distinct activation. Artistic facial expressions of pain and non-artistic neutral facial expressions trigger the response of the rIOG. This region belongs to the distributed neural system devoted to face perception (Haxby *et al.*, 2002; Rossion *et al.*, 2012). The cluster here identified includes the occipital face area, a functionally defined face-selective area usually located in the lateral surface of the occipital lobe either in or in the vicinity of the IOG (Pitcher *et al.*, 2011; Rossion *et al.*, 2012). Even if under debate, the rIOG is described as the first cortical relay of face processing contributing to an early structural description of the face (Haxby *et al.*, 2002; Rossion *et al.*, 2012). The activation of the rIOG in response to artistic facial expressions of pain and non-artistic neutral faces may suggest that the decoding of such stimuli takes advantage from a detailed analysis of facial visual proprieties.

A number of specific methodological choices were made in our paradigm. Here, a balance between a rigorous control over stimuli properties and the ecological power of artistic images was obtained through a meticulous and hypothesis-driven procedure of stimuli selection and validation, potentially limiting the study’s general validity. Despite this rigorous procedure, some differences between artistic and non-artistic stimuli remain (i.e. realism rating). The artistic images used in empirical studies interested in art are not created for experimental purposes and therefore possess a variety of elements that cannot be fully controlled, which are congenital to the artistic nature of the images themselves. However, the presence of these differences requires caution in the interpretation and generalization of our results. Due to the specificity of our stimuli, we selected a small number of images that consequently required a relatively high number of repetitions (*n* = 6) across the experimental runs, potentially leading to a decrement in Blood Oxygenation Level Dependent (BOLD) activation. Participants made continuous AJs on stimuli categorized dichotomously as artistic or non-artistic. This procedure constitutes a necessary mismatch between the theoretical formulation and the methodological choices aimed to measure the aesthetic experience (see the ‘Introduction’ section). However, it is important to note that only the judgements offered to artistic images were entered in the ROIs analyses to respond to the hypotheses formulated. With respect to the procedure followed in this study, participants always performed the pain identification task inside the scanner. This procedure was followed in agreement with the extensive literature on empathy for pain adopting the same paradigm, but it prevents us from establishing that the same activation patterns are also possible during the ‘task-free’ observation of pictorial pain expressions. Further studies are needed to investigate the neural activations associated with the AJ of artistic facial expressions of pain, to support and extend the present results. Although observation of painful facial expressions engages pain-related regions (i.e. AI, pACC and aMCC), several arguments have been raised about the pain specificity of those areas, with critical implications for the functional interpretation of the neural overlap triggered by experiencing one’s own pain and observing others’ pain (Iannetti *et al.*, 2013; Zaki *et al.*, 2016). Lastly, whereas we were able to formulate specific predictions about the results expected for our first two aims, our third goal was mostly explorative. In this case, we can only suggest cautious interpretations, accounting for the notion of reverse inference.

In conclusion, our results show that empathy for pain brain regions can also be activated by artistic pain representations. Correlational analyses between functional brain responses and aesthetic ratings suggest that the activity of the insular and cingulate cortices concurs with the formation of an AJ in non-art expert observers. This work supports the necessity to further investigate how non-beauty-specific neural mechanisms could feed the complex phenomena of aesthetic experience and AJ.

## Supplementary Material

nsab066_Supp

## References

[R1] Ardizzi M. (2020). Una prospettiva integrata per un’estetica empirica. *Giornale Italiano Di Psicologia*, 47, 95–100.

[R2] Ardizzi M. , Ferroni F., Siri F., et al. (2020a). Beholders’ sensorimotor engagement enhances aesthetic rating of pictorial facial expressions of pain. *Psychological Research*, 84, 370–9. 30073408 10.1007/s00426-018-1067-7

[R3] Ardizzi M. , Calbi M., Tavaglione S., et al. (2020b). Audience spontaneous entrainment during the collective enjoyment of live performances: physiological and behavioral measurements. *Scientific Reports*, 10, 1–12. 32123246 10.1038/s41598-020-60832-7 PMC7052145

[R0004a] Bachrach A. , Jola C, Pallier C. (2016). Neuronal bases of structural coherence in contemporary dance observation. *NeuroImage*, 124, 464–72. 26348557 10.1016/j.neuroimage.2015.08.072

[R4] Battaglia F. , Lisanby S.H., Freedberg D. (2011). Corticomotor excitability during observation and imagination of a work of art. *Frontiers in Human Neuroscience*, 5, 79. 10.3389/fnhum.2011.00079 PMC3159953 21897813

[R5] Benuzzi F. , Lui F., Ardizzi M., et al. (2018). Pain mirrors: neural correlates of observing self or others’ facial expressions of pain. *Frontiers in Psychology*, 9, 1825. 10.3389/fpsyg.2018.01825 PMC6175971 30333771

[R6] Botvinick M. , Jha A.P., Bylsma L.M., et al. (2005). Viewing facial expressions of pain engages cortical areas involved in the direct experience of pain. *NeuroImage*, 25, 312–9. 15734365 10.1016/j.neuroimage.2004.11.043

[R7] Budell L. , Jackson P., Rainville P. (2010). Brain responses to facial expressions of pain: emotional or motor mirroring? *NeuroImage*, 53, 355–63. 20510372 10.1016/j.neuroimage.2010.05.037

[R8] Budell L. , Kunz M., Jackson P.L., et al. (2015). Mirroring pain in the brain: emotional expression versus motor imitation. *PLoS One*, 10, e0107526. 10.1371/journal.pone.0107526 PMC4324963 25671563

[R9] Calvo-Merino B. , Jola C., Glaser D.E., Haggard P. (2008). Towards a sensorimotor aesthetics of performing art. *Consciousness and Cognition*, 17, 911–22. 18207423 10.1016/j.concog.2007.11.003

[R10] Carr L. , Iacoboni M., Dubeau M.-C., et al. (2003). Neural mechanisms of empathy in humans: a relay from neural systems for imitation to limbic areas. *Proceedings of the National Academy of Sciences of the United States of America*, 100, 5497–502. 12682281 10.1073/pnas.0935845100 PMC154373

[R11] Carrillo M. , Han Y., Migliorati F., et al. (2019). Emotional mirror neurons in the rat’s anterior cingulate cortex. *Current Biology*, 29, 1301–12.e6. 30982647 10.1016/j.cub.2019.03.024 PMC6488290

[R12] Caruana F. , Avanzini P., Gozzo F., et al. (2017). A mirror mechanism for smiling in the anterior cingulate cortex. *Emotion*, 17, 187–90. 27854442 10.1037/emo0000237

[R13] Caruana F. , Gerbella M., Avanzini P., et al. (2018). Motor and emotional behaviours elicited by electrical stimulation of the human cingulate cortex. *Brain*, 141, 3035–51. 30107501 10.1093/brain/awy219

[R14] Cattaneo Z. , Lega C., Flexas A., et al. (2014). The world can look better: enhancing beauty experience with brain stimulation. *Social Cognitive and Affective Neuroscience*, 9, 1713–21. 24132459 10.1093/scan/nst165 PMC4221210

[R15] Cattaneo Z. , Schiavi S., Silvanto J., et al. (2015). A TMS study on the contribution of visual area V5 to the perception of implied motion in art and its appreciation. *Cognitive Neuroscience*, 8, 59–68. 26429631 10.1080/17588928.2015.1083968

[R16] Cavanna A.E. , Trimble M.R. (2006). The precuneus: a review of its functional anatomy and behavioural correlates. *Brain*, 129, 564–83. 16399806 10.1093/brain/awl004

[R17] Cela-Conde C.J. , Marty G., Maestú F., et al. (2004). Activation of the prefrontal cortex in the human visual aesthetic perception. *Proceedings of the National Academy of Sciences of the United States of America*, 101, 6321–5. 15079079 10.1073/pnas.0401427101 PMC395967

[R18] Chatterjee A. , Widick P., Sternschein R., Smith W.B., Bromberger B. (2010). The assessment of art attributes. *Empirical Studies of the Arts*, 28, 207–22.

[R19] Chatterjee A. , Vartanian O. (2016). Neuroscience of aesthetics. *Annals of the New York Academy of Sciences*, 1369, 172–94. 27037898 10.1111/nyas.13035

[R20] Cohen A.L. , Soussand L., Corrow S.L., et al. (2019). Looking beyond the face area: lesion network mapping of prosopagnosia. *Brain*, 142, 3975–90. 31740940 10.1093/brain/awz332 PMC6906597

[R21] De Gelder B. , Watson R., Zhan M., et al. (2018). Classical paintings may trigger pain and pleasure in the gendered brain. *cortex*, 109, 171–80. 30388438 10.1016/j.cortex.2018.09.011

[R22] Di Dio C. , Ardizzi M., Massaro D., et al. (2016). Human, nature, dynamism: the effects of content and movement perception on brain activations during the aesthetic judgment of representational paintings. *Frontiers in Human Neuroscience*, 9, 1–19. 10.3389/fnhum.2015.00705 PMC4709505 26793087

[R23] Duff E.P. , Cunnington R., Egan G.F. (2007). REX: response exploration for neuroimaging datasets. *Neuroinformatics*, 5, 223–34. 17985253 10.1007/s12021-007-9001-y

[R24] Ebisch S.J.H. , Perrucci M.G., Ferretti A., et al. (2008). The sense of touch: embodied simulation in a visuotactile mirroring mechanism for observed animate or inanimate touch. *Journal of Cognitive Neuroscience*, 20, 1611–23. 18345991 10.1162/jocn.2008.20111

[R25] Ebisch S.J.H. , Ferri F., Salone A., et al. (2011). Differential involvement of somatosensory and interoceptive cortices during the observation of affective touch. *Journal of Cognitive Neuroscience*, 23, 1808–22. 20666597 10.1162/jocn.2010.21551

[R26] Eickhoff S.B. , Stephan K.E., Mohlberg H., et al. (2005). A new SPM toolbox for combining probabilistic cytoarchitectonic maps and functional imaging data. *NeuroImage*, 25, 1325–35. 15850749 10.1016/j.neuroimage.2004.12.034

[R27] Fiori F. , Plow E., Rusconi M.L., Cattaneo Z. (2020). Modulation of corticospinal excitability during paintings viewing: a TMS study. *Neuropsychologia*, 149, 107664. 10.1016/j.neuropsychologia.2020.107664 33130160

[R28] Freedberg D. , Gallese V. (2007). Motion, emotion and empathy in esthetic experience. *Trends in Cognitive Sciences*, 11, 197–203. 17347026 10.1016/j.tics.2007.02.003

[R29] Friston K.J. , Holmes A.P., Worsley K.J. (1999). How many subjects constitute a study? *NeuroImage*, 10, 1–5. 10385576 10.1006/nimg.1999.0439

[R30] Friston K.J. , Glaser D.E., Henson R.N.A., et al. (2002). Classical and Bayesian inference in neuroimaging: applications. *NeuroImage*, 16, 484–512. 12030833 10.1006/nimg.2002.1091

[R31] Gallese V. , Keysers C., Rizzolatti G. (2004). A unifying view of the basis of social cognition. *Trends in Cognitive Sciences*, 8, 396–403. 15350240 10.1016/j.tics.2004.07.002

[R32] Gallese V. (2014). Bodily selves in relation: embodied simulation as second-person perspective on intersubjectivity. *Philosophical Transactions of the Royal Society B: Biological Sciences*, 369, 20130177. 10.1098/rstb.2013.0177 PMC4006180 24778374

[R33] Gallese V. (2017). Visions of the body. Embodied simulation and aesthetic experience. *Aisthesis*, 10, 41–50.

[R34] Gallese V. , Sinigaglia C. (2011). What is so special about embodied simulation? *Trends in Cognitive Sciences*, 15, 512–9. 21983148 10.1016/j.tics.2011.09.003

[R35] Gu X. , Gao Z., Wang X., et al. (2012). Anterior insular cortex is necessary for empathetic pain perception. *Brain*, 135, 2726–35. 22961548 10.1093/brain/aws199 PMC3437027

[R36] Haxby J.V. , Hoffman E.A., Gobbini M.I. (2002). Human neural systems for face recognition and social communication. *Biological Psychiatry*, 51, 59–67. 11801231 10.1016/s0006-3223(01)01330-0

[R37] Hayn-Leichsenring G.U. (2017). The ambiguity of artworks - a guideline for empirical aesthetics research with artworks as stimuli. *Frontiers in Psychology*, 8, 1–14. 29123494 10.3389/fpsyg.2017.01857 PMC5662902

[R38] Hennenlotter A. , Schroeder U., Erhard P., et al. (2005). A common neural basis for receptive and expressive communication of pleasant facial affect. *NeuroImage*, 26, 581–91. 15907315 10.1016/j.neuroimage.2005.01.057

[R39] Iannetti G.D. , Salomons T.V., Moayedi M., et al. (2013). Beyond metaphor: contrasting mechanisms of social and physical pain. *Trends in Cognitive Sciences*, 17, 371–8. 23796880 10.1016/j.tics.2013.06.002

[R40] Ishizu T. (2014). A neurobiological enquiry into the origins of our experience of the sublime and beautiful. *Frontiers in Human Neuroscience*, 8, 1–10. 25426046 10.3389/fnhum.2014.00891 PMC4227571

[R41] Ishizu T. , Zeki S. (2011). Toward a brain-based theory of beauty. *PLoS One*, 6, e21852. 10.1371/journal.pone.0021852 PMC3130765 21755004

[R42] Ishizu T. , Zeki S. (2017). The experience of beauty derived from sorrow. *Human Brain Mapping*, 38, 4185–200. 28544456 10.1002/hbm.23657 PMC5518297

[R43] Jabbi M. , Keysers C. (2008). Inferior frontal gyrus activity triggers anterior insula response to emotional facial expressions. *Emotion*, 8, 775–80. 19102588 10.1037/a0014194

[R44] Kaltwasser L. , Rost N., Ardizzi M., et al. (2019). Sharing the filmic experience-the physiology of socio-emotional processes in the cinema. *PLoS One*, 14, e0223259. 10.1371/journal.pone.0223259 PMC6799930 31626656

[R45] Kirsch L.P. , Urgesi C., Cross E.S. (2016). Shaping and reshaping the aesthetic brain: emerging perspectives on the neurobiology of embodied aesthetics. *Neuroscience and Biobehavioral Reviews*, 62, 56–68. 26698020 10.1016/j.neubiorev.2015.12.005

[R46] Koelsch S. (2014). Brain correlates of music-evoked emotions. *Nature Reviews Neuroscience*, 15, 170–80. 24552785 10.1038/nrn3666

[R47] Kurth F. , Zilles K., Fox P.T., et al. (2010). A link between the systems: functional differentiation and integration within the human insula revealed by meta-analysis. *Brain Structure & Function*, 214, 519–34. 20512376 10.1007/s00429-010-0255-z PMC4801482

[R48] Labek K. , Berger S., Buchheim A., et al. (2017). The iconography of mourning and its neural correlates: a functional neuroimaging study. *Social Cognitive and Affective Neuroscience*, 12, 1303–13. 28449116 10.1093/scan/nsx058 PMC5597887

[R49] Lamm C. , Decety J., Singer T. (2011). Meta-analytic evidence for common and distinct neural networks associated with directly experienced pain and empathy for pain. *NeuroImage*, 54, 2492–502. 20946964 10.1016/j.neuroimage.2010.10.014

[R50] Leder H. , Bär S., Topolinski S. (2012). Covert painting simulations influence aesthetic appreciation of artworks. *Psychological Science*, 23, 1479–81. 23137968 10.1177/0956797612452866

[R51] Lundqvist L.O. , Carlsson F., Hilmersson P., et al. (2009). Emotional responses to music: experience, expression, and physiology. *Psychology of Music*, 37, 61–90.

[R52] McLean C. , Want S.C., Dyson B.J. (2015). The role of similarity, sound and awareness in the appreciation of visual artwork via motor simulation. *Cognition*, 137, 174–81. 25659540 10.1016/j.cognition.2015.01.002

[R53] Molenberghs P. , Johnson H., Henry J.D., Mattingley J.B. (2016). Understanding the minds of others: a neuroimaging meta-analysis. *Neuroscience and Biobehavioral Reviews*, 65, 276–91. 27073047 10.1016/j.neubiorev.2016.03.020

[R54] Oldfield R.C. (1971). The assessment and analysis of handedness: the Edinburgh inventory. *Neuropsychologia*, 9, 97–113. 5146491 10.1016/0028-3932(71)90067-4

[R55] Ostrowsky K. , Isnard J., Ryvlin P., et al. (2000). Functional mapping of the insular cortex: clinical implication in temporal lobe epilepsy. *Epilepsia*, 41, 681–6. 10840399 10.1111/j.1528-1157.2000.tb00228.x

[R56] Ostrowsky K. , Magnin M., Ryvlin P., et al. (2002). Representation of pain and somatic sensation in the human insula: a study of responses to direct electrical cortical stimulation. *Cerebral Cortex (New York, NY: 1991)*, 12, 376–85. 10.1093/cercor/12.4.376 11884353

[R57] Pearce M.T. , Zaidel D.W., Vartanian O., et al. (2016). Neuroaesthetics: the cognitive neuroscience of aesthetic experience. *Perspectives on Psychological Science*, 11, 265–79. 26993278 10.1177/1745691615621274

[R58] Pelowski M. , Markey P.S., Forster M., Gerger G., Leder H. (2017). Move me, astonish me… delight my eyes and brain: the Vienna integrated model of top-down and bottom-up processes in art perception (VIMAP) and corresponding affective, evaluative, and neurophysiological correlates. *Physics of Life Reviews*, 21, 80–125. 28347673 10.1016/j.plrev.2017.02.003

[R59] Pitcher D. , Walsh V., Duchaine B. (2011). The role of the occipital face area in the cortical face perception network. *Experimental Brain Research*, 209, 481–93. 21318346 10.1007/s00221-011-2579-1

[R60] Rizzolatti G. , Sinigaglia C. (2016). The mirror mechanism: a basic principle of brain function. *Nature Reviews Neuroscience*, 17, 757–65. 27761004 10.1038/nrn.2016.135

[R61] Rossion B. , Hanseeuw B., Dricot L. (2012). Defining face perception areas in the human brain: a large-scale factorial fMRI face localizer analysis. *Brain and Cognition*, 79, 138–57. 22330606 10.1016/j.bandc.2012.01.001

[R62] Schott G.D. (2015). Pictures of pain: their contribution to the neuroscience of empathy. *Brain*, 138, 812–20. 25614024 10.1093/brain/awu395 PMC4408436

[R0063a] Schurz M. , Radua J., Aichhorn M., Richlan F., Perner J. (2014). Fractionating theory of mind: a meta-analysis of functional brain imaging studies. *Neuroscience & Biobehavioral Reviews*, 42, 9–34. 24486722 10.1016/j.neubiorev.2014.01.009

[R63] Shamay-Tsoory S.G. , Aharon-Peretz J., Perry D. (2009). Two systems for empathy: a double dissociation between emotional and cognitive empathy in inferior frontal gyrus versus ventromedial prefrontal lesions. *Brain*, 132, 617–27. 18971202 10.1093/brain/awn279

[R64] Siri F. , Ferroni F., Ardizzi M., et al. (2018). Behavioral and autonomic responses to real and digital reproductions of works of art. *Progress in Brain Research*, 237, 201–21. 29779735 10.1016/bs.pbr.2018.03.020

[R65] Thakral P.P. , Moo L.R., Slotnick S.D. (2012). A neural mechanism for aesthetic experience. *NeuroReport*, 23, 310–3. 22357395 10.1097/WNR.0b013e328351759f

[R66] Ticini L.F. , Rachman L., Pelletier J., et al. (2014). Enhancing aesthetic appreciation by priming canvases with actions that match the artist’s painting style. *Frontiers in Human Neuroscience*, 8, 1–6. 24917808 10.3389/fnhum.2014.00391 PMC4043134

[R67] van der Gaag C. , Minderaa R.B., Keysers C. (2007). Facial expressions: what the mirror neuron system can and cannot tell us. *Social Neuroscience*, 2, 179–222. 18633816 10.1080/17470910701376878

[R68] Wassiliwizky E. , Koelsch S., Wagner V., et al. (2017). The emotional power of poetry: neural circuitry, psychophysiology and compositional principles. *Social Cognitive and Affective Neuroscience*, 12, 1229–40. 28460078 10.1093/scan/nsx069 PMC5597896

[R69] Weisholtz D.S. , Root J.C., Butler T., et al. (2015). Beyond the amygdala: linguistic threat modulates peri-sylvian semantic access cortices. *Brain and Language*, 151, 12–22. 26575986 10.1016/j.bandl.2015.10.004 PMC4743641

[R70] Wicker B. , Keysers C., Plailly J., et al. (2003). Both of us disgusted in My insula: the common neural basis of seeing and feeling disgust. *Neuron*, 40, 655–64. 14642287 10.1016/s0896-6273(03)00679-2

[R71] Zaki J. , Wager T.D., Singer T., et al. (2016). The anatomy of suffering: understanding the relationship between nociceptive and empathic pain. *Trends in Cognitive Sciences*, 20, 249–59. 26944221 10.1016/j.tics.2016.02.003 PMC5521249

[R72] Zeki S. , Romaya J.P., Benincasa D.M.T., et al. (2014). The experience of mathematical beauty and its neural correlates. *Frontiers in Human Neuroscience*, 8, 1–12. 24592230 10.3389/fnhum.2014.00068 PMC3923150

