# Supplementary material for: Visceromotor roots of aesthetic evaluation of pain in art: an fMRI study
Source: Soc Cogn Affect Neurosci. 2021 May 14;16(11):1113–22. doi: 10.1093/scan/nsab066 (PMC8599194; doi:10.1093/scan/nsab066)
Supplement: nsab066_Supp [file nsab066_supp.zip › Supplementary Material.docx]

**Supplementary Material of “Visceromotor roots of aesthetic evaluation of pain in art: an fMRI study” by**

**Ardizzi Martina, Ferroni Francesca, Umiltà Maria Alessandra, Pinardi Chiara, Errante Antonino, Ferri Francesca, Fadda Elisabetta and Gallese Vittorio**

**Images selection and stimuli validation**

Paintings depicting artistic painful facial expressions were identified choosing those artworks in which the artist’s intent was to represent scenes of physical pain. Depictions of Jesus Christ were excluded to avoid contextual bias. Paintings depicting artistic neutral facial expressions were selected among portraits of the same historical period. Non-artistic images represent facial expressions of volunteers who posed for a short video-clip. We chose posed images, rather than spontaneous expressions of pain obtained from a real nociceptive stimulation, to balance as much as possible the presence of a communicative intentionality between non-artistic and artistic stimuli. The latter in fact arise from the artist’s explicit and conscious intention to communicate such adverse physical condition which could be missing in a spontaneous reaction to a noxious stimulus. A video camera (Sony HDD handycam DCR-SR32, spatial resolution 720 x 576 pixels) was mounted on a tripod about 1.5 m in front of the participants, at the eye level. Environmental setting (e.g., room light, camera distance, sitting position) was kept constant. Non-artistic facial expressions of pain were obtained asking models to imagine feeling a severe physic pain (e.g., “Imagine having broken your leg”). The resulting video-frame representing the apex of the painful facial expressions was selected. Conversely, non-artistic neutral images were obtained asking volunteers to observe a fixation point in front of them trying to maintain a neutral facial expression as if they were posing for a portrait. Only male facial expressions were selected because a pilot images selection demonstrated how artistic female facial expressions of pain were not correctly recognized when other pictorial contextual features were removed. These findings are coherent with art literature showing that in most artistic representations of pain the faces of the victims experiencing pain were depicted without the expected expression, especially in the case of women (Moscoso et al., 2012; Schott, 2015). Digital versions of the images were modified by means of Adobe Photoshop CC2015 software. The images resolution was adjusted at 300 pixels/pitch. Only the face was selected removing all other contextual variables and presented against a uniform grey background of 600x800 pixels. The dimension and orientation of the selected faces were uniformed.

Following the here above-described procedure, a total of 50 images were obtained.

In order to select the 24 stimuli used for the present experiment, images were submitted to a pre-experimental validation phase. Twenty volunteers (10 females; mean age=24.7 years, SD=3.40), untutored in art or art history and not subsequently involved in the experiment, were asked to judge images overall emotional (i.e., “How much emotionally intense is this image?”), pain intensity (i.e., “How much pain does this image express?”) and realism (i.e., “How much realistic is this image?”) using a 5-point ordinal scale ranging from ‘not at all’ (0) to ‘extremely emotional/painful’ (4). Painful and neutral facial expressions were then selected, separately for artistic and non-artistic images, according to the following criteria: I) painful facial expressions (n.6 Art Pain and n.6 Non-Art Pain) were selected among those images obtaining the highest evaluation for pain intensity; II) neutral facial expressions (n.6 Art Neutral and n.6 Non-Art Neutral) were selected among those images obtaining the lowest emotional evaluation.

To check the equivalence between artistic and non-artistic selected images, a repeated-measures ANOVA was conducted including Content (Art, Non-Art), Emotion (Pain, Neutral) and Question (Pain Intensity, Emotional Intensity, Realism) as within factors. The interaction Content by Emotion by Question was significant (F_(2,10)_=6.746, p=0.014). Post-hoc comparisons, using Bonferroni adjustment, demonstrated no significant difference between Art Pain and Non-Art Pain stimuli in pain and emotional intensity judgments (Pain intensity: Art Pain= 2.33, SE 0.16; Non-Art Pain= 2.07, SE 0.08; p = 0.06; Emotional intensity: Art Pain= 2.5, SE 0.12; Non-Art Pain= 2.33, SE 0.1; p = 0.063). Differently, the realism of Art Pain stimuli was rated significantly higher than non-Art Pain stimuli (Art Pain: 2.26, SE 0.17; Non-Art Pain: 1.80, SE 0.11; p = 0.0005). The higher realism observed for Artistic stimuli can reasonably be attributed to the creative competence of the artist (absent in Non-Artistic stimuli). No significant differences were found between Art Neutral and Non-Art Neutral stimuli for all judgments (Pain intensity: Art Neutral= 0.5, SE 0.04; Non-Art Neutral= 0.48, SE 0.08; p = 1; Emotional intensity: Art Neutral= 1.19, SE 0.09; Non-Art Neutral= 0.99, SE 0.03; p = 0.40; Realism: Art Neutral= 2.04, SE 0.06; Non-Art Neutral= 2.13, SE 0.08; p = 1).

The brightness of Artistic and Non-Artistic stimuli was not different (Art Pain: 110.50 cd/m^2^, SE 2.87; Art Neutral: 128.93 cd/m^2^, SE 4.68; Non-Art Pain: 110.43 cd/m^2^, SE 5; Non-Art Neutral: 134.87 cd/m^2^, SE 3.31; F_(1,5)_=0.858, p=0.397). Refer to Supplementary Figure 1 and Supplementary Table 1 for the final set images selected.

In order to check for the equivalence in terms of both valence and intensity ratings between artistic and non-artistic selected images, a supplementary post-hoc validation of the stimuli was conducted in a new sample of 20 participants. Twenty volunteers (12 females; mean age= 28 years, SE=1.27), untutored in art or art history and not involved in the previous validation or the main experiment, were asked to judge images valence (i.e., “How would you judge the valence of the expressed emotion of this image?”) and emotional intensity (i.e., “How much emotionally intense would you judge this image?”). In order to answer to these two questions, participants used 5-point ordinal scale ranging from ‘very negative/calm’ (0) to ‘very positive/excited’ (4), respectively. Then, participants’ ratings were entered into a repeated-measures ANOVA, with Content (Art, Non-Art), Emotion (Pain, Neutral) and Question (Valence, Intensity) as within-subjects factors. The main effect of Emotion (F_(1,19)_= 20.06, p<0.001; η^2^_p_ =0.51) resulted significant, showing higher ratings for painful facial expressions than for neutral ones (Pain: 3.02 SE 0.02; Neutral: 2.77 SE 0.04). The interaction Emotion by Question resulted significant (F_(1,19)_= 586.04, p<0.001; η^2^_p_ = 0.97). Bonferroni post-hoc comparisons demonstrated differences between painful and neutral facial expressions in terms of both emotional intensity and valence (Intensity Pain: 4.46 SE 0.06; Intensity Neutral: 2.59 SE 0.08; p < 0.001; Valence Pain 1.57 SE 0.07; Valence Neutral: 2.95 SE 0.04, p < 0.001). Indeed, the emotional intensity of painful stimuli was rated significantly higher than neutral stimuli whereas the valence of neutral stimuli was rated higher than painful ones. No differences were found between artistic and non-artistic stimuli.

**Supplementary Table 1 – List of selected artistic stimuli.**

|  | **Author** | **Title** | **Collocation** | **Year** |  |
| --- | --- | --- | --- | --- | --- |
| Art Pain Stimuli | Caravaggio | David and Goliath | Borghese Gallery (Rome) | 1609-1610 | 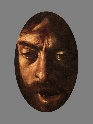 |
|  | Theodoor Rombouts | Prometheus | Royal museum of Fine Arts of Belgium (Antwerp) | 1590-1596 | 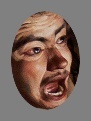 |
|  | Giudo Reni | Apollo and Marsia | Musée des Augustins (Tolosa) | 1618-1619 | 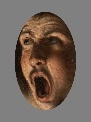 |
|  | Gregorio Martinez | Prometheus bound | The Prado Museum (Madrid) | 1590-1596 | 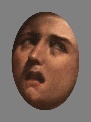 |
|  | Josè de Ribera | Martyrdom of Saint Philip | The Prado Museum (Madrid) | 1639 | 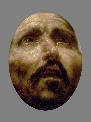 |
|  | Cerano | Martyrdom of Saint Bartholomeus | Tacchi-Venturi Art Gallery (San Severino) | 1630 | 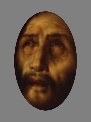 |
| Art Neutral Stimuli | Lorenzo Lotto | Portrait of a Gentleman in his study | Academic Gallery (Venice) | 1535 | 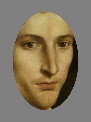 |
|  | Antonello da Messina | Portrait of a Man | National Gallery (London) | 1476 | 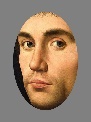 |
|  | Annibale Carracci | Self-portrait | Guelpa Foundation (Turin) | 1593 | 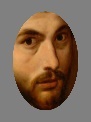 |
|  | Sandro Botticelli | Portrait of a Young Man | National Gallery (London) | 1483-1484 | 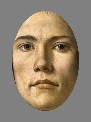 |
|  | Hans Memling | Portrait of a Man with a Roman Coin | Royal museum of Fine Arts of Belgium (Antwerp) | 1480 | 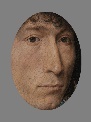 |
|  | Théodore Géricault | Portrait of Eugene Delacroix | Musée des Beaux-Arts (Rouen) | 1818-1819 | 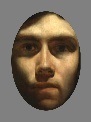 |

**
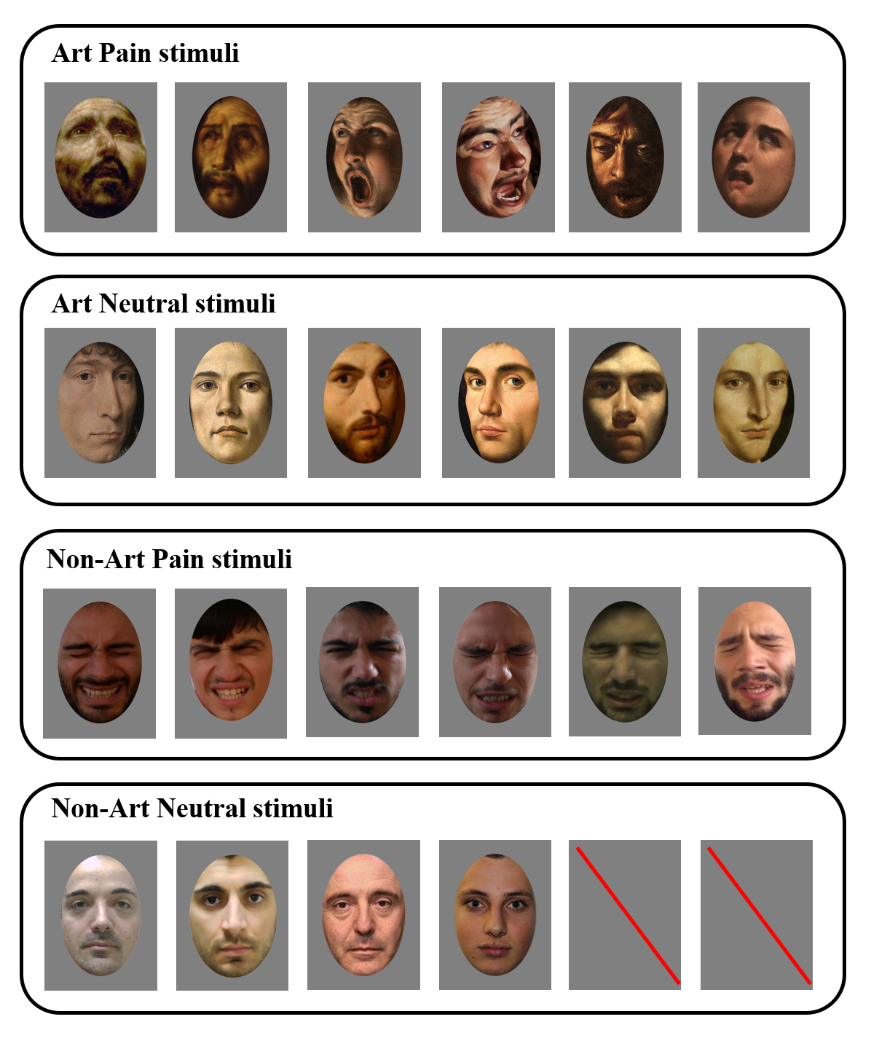
**

**Supplementary Fig. 1. Experimental stimuli used in the experimental protocol.** Stimuli crossed out in red are stimuli for which there is no consent to be published.

**Supplementary functional results – Whole brain activations vs. Baseline**

The activation patterns observed for each stimulus-category (AP, AN, nAP, nAN) vs. implicit baseline (fixation cross) were thresholded at p < 0.05 family wise error (FWE) corrected at the cluster level (cluster size estimated with a voxel-level threshold at p < 0.05 FWE). The activation patterns observed for each stimulus-category vs. implicit baseline were very similar. Not surprisingly, activations were observed in a large occipital cluster encompassing the fusiform face area, in the left hippocampus and the right thalamus. These structures are involved in the perceptual analysis, implicit memory integration and explicit classification of the stimuli (Leder et al., 2017). Sensorimotor cortices, including the precentral gyrus, were also ubiquitously activated. The recruitment of such sensorimotor circuits could be associated to eye-movements, as common in studies that allow free exploration of stimuli but that use fixation cross during baseline periods (e.g., Di Dio et al., 2016). We refer to the main text for a further discussion of activation patterns better explained by specific main effects and interaction between stimulus-categories. Please see Supplementary Figure 2 and Supplementary Table 2 for a detailed results description.

**
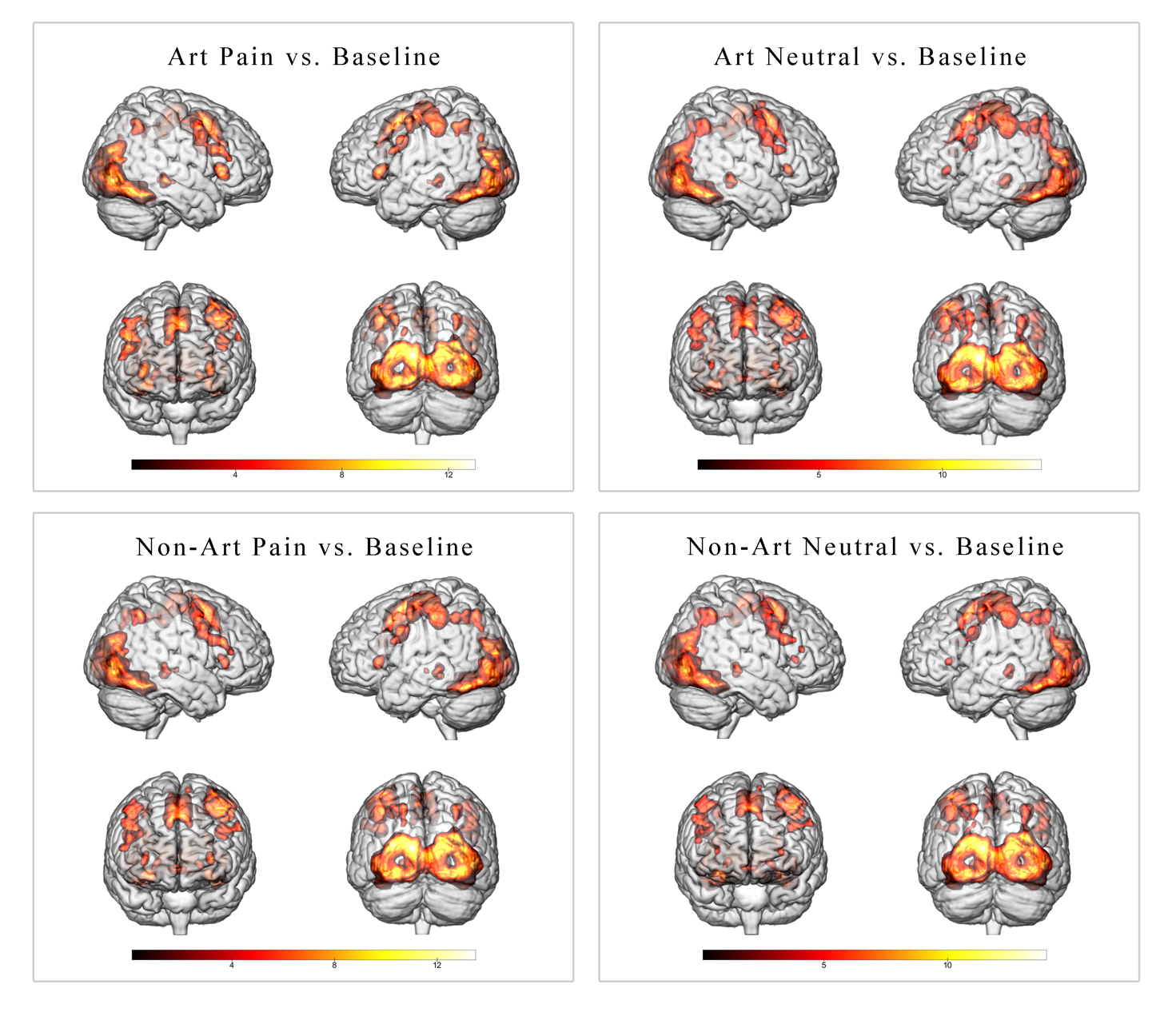
Supplementary Fig. 2. Brain activation maps observed for each stimulus-category (AP, AN, nAP, nAN) vs. baseline.** Group-averaged statistical parametric maps are rendered into a standard MNI brain template (P FWE corr < 0.05). Colour bars show t-values.

**Supplementary Table 2 – Contrast analyses for each stimulus-category vs. baseline**

| **Contrast** | **Brain structure** | **Side** | **Ke** | **p. FWE corr  cluster level** | **Z** | **Local Maxima (MNI)** | | |
| --- | --- | --- | --- | --- | --- | --- | --- | --- |
|  |  |  |  |  |  | **x** | **y** | **z** |
| **AP vs. Baseline** | Fusiform Gyrus  Middle Occipital Gyrus | R/L | 58321 | 0.001 | Inf | 36 | -52 | -16 |
|  |  |  |  |  | Inf | -23 | -77 | -11 |
|  |  |  |  |  | Inf | -30 | -87 | 15 |
|  | Posterior-Medial Frontal  Anterior Cingulate Cortex  Middle Cingulate Cortex | R/L | 9092 | 0.001 | Inf | -5 | 6 | 54 |
|  |  |  |  |  | Inf | 7 | 9 | 51 |
|  |  |  |  |  | 7.31 | 7 | 15 | 43 |
|  | Inferior pariental lobule  Precentral Gyrus | L | 10502 | 0.001 | 7.42 | -41 | -32 | 49 |
|  |  |  |  |  | 7.3 | -36 | -21 | 53 |
|  |  |  |  |  | 7.14 | -37 | -17 | 65 |
|  | Inferior Frontal gyrus  Middle Frontal gyrus | R | 4406 | 0.001 | 7.02 | 44 | 5 | 34 |
|  |  |  |  |  | 6.74 | 44 | 18 | 22 |
|  |  |  |  |  | 5.91 | 44 | 2 | 45 |
|  | Anterior Insula | R | 1285 | 0.001 | 6.86 | 31 | 26 | 5 |
|  | Inferior Frontal gyrus | L | 853 | 0.001 | 6.7 | -29 | 26 | 4 |
|  | Hippocampus | R | 379 | 0.001 | 6.5 | 22 | -28 | -4 |
|  | Hippocampus | L | 318 | 0.001 | 6.43 | -19 | -31 | -3 |
|  | Inferior pariental lobule | L | 876 | 0.001 | 6.3 | -28 | -56 | 50 |
|  |  |  |  |  | 5.9 | -31 | -47 | 47 |
|  |  |  |  |  | 5.04 | -29 | -48 | 39 |
|  | Inferior Frontal gyrus | L | 1129 | 0.001 | 6.02 | -41 | 3 | 30 |
|  |  |  |  |  | 5.65 | -40 | 10 | 24 |
|  | Precentral Gyrus | L | 160 | 0.001 | 5.74 | -57 | 5 | 34 |
|  | Inferior pariental lobule | R | 813 | 0.001 | 5.74 | 31 | -55 | 50 |
|  | Superior Occipital Gyrus | L | 127 | 0.001 | 5.37 | -18 | -70 | 35 |
| **AN vs. Baseline** | Fusiform Gyrus  Cerebellum | R/L | 82289 | 0.001 | Inf | 36 | -52 | -16 |
|  |  |  |  |  | Inf | 21 | -77 | -12 |
|  |  |  |  |  | Inf | -22 | -77 | -11 |
|  | Posterior-Medial Frontal gyrus  Middle Cingulate Cortex | R/L | 10482 | 0.001 | Inf | -5 | 5 | 54 |
|  |  |  |  |  | Inf | 7 | 8 | 51 |
|  |  |  |  |  | 7.29 | 8 | 12 | 44 |
|  | Hippocampus | L | 517 | 0.001 | 6.88 | -20 | -31 | -3 |
|  | Inferior Frontal gyrus  Precentral Gyrus | R | 3788 | 0.001 | 6.67 | 44 | 5 | 33 |
|  |  |  |  |  | 5.89 | 43 | 1 | 46 |
|  |  |  |  |  | 5.81 | 44 | 17 | 23 |
|  | Thalamus | R | 441 | 0.001 | 6.48 | 21 | -29 | -2 |
|  | Inferior Frontal gyrus  Precentral Gyrus | L | 813 | 0.001 | 5.87 | -41 | 3 | 30 |
|  |  |  |  |  | 5.85 | -57 | 5 | 34 |
|  |  |  |  |  | 4.87 | -39 | 9 | 24 |
|  | Inferior Frontal gyrus | L | 240 | 0.001 | 5.84 | -29 | 27 | 4 |
|  | Anterior Insula | R | 228 | 0.001 | 5.59 | 31 | 25 | 6 |
| **nAP vs. Baseline** | Fusiform Gyrus  Middle Occipital Gyrus | R/L | 59351 | 0.001 | Inf | -21 | -86 | 16 |
|  |  |  |  |  | Inf | 27 | -78 | 18 |
|  |  |  |  |  | Inf | 36 | -52 | -16 |
|  | Posterior-medial Frontal gyrus  Middle Cingulate Cortex | R/L | 10455 | 0.001 | Inf | -5 | 5 | 54 |
|  |  |  |  |  | Inf | 7 | 8 | 51 |
|  |  |  |  |  | 7.41 | 8 | 13 | 43 |
|  | Precentral Gyrus  Inferior pariental lobule | L | 14970 | 0.001 | Inf | -36 | -21 | 53 |
|  |  |  |  |  | 7.7 | -37 | -18 | 65 |
|  |  |  |  |  | 7.59 | -40 | -32 | 48 |
|  | Inferior Frontal gyrus  Middle Frontal Gyrus | R/L | 5495 | 0.001 | 6.6 | 44 | 5 | 33 |
|  |  |  |  |  | 6.35 | 44 | 17 | 23 |
|  |  |  |  |  | 6.1 | 43 | 2 | 45 |
|  | Inferior Frontal gyrus | L | 498 | 0.001 | 6.35 | -29 | 27 | 3 |
|  | Inferior Frontal gyrus  Precentral Gyrus | L | 1738 | 0.001 | 6.31 | -41 | 3 | 30 |
|  |  |  |  |  | 6.16 | -58 | 6 | 33 |
|  | Inferior pariental lobule | R | 1080 | 0.001 | 6.22 | 32 | -54 | 49 |
|  | Hippocampus | L | 291 | 0.001 | 6.16 | -19 | -31 | -2 |
|  | Anterior Insula | R | 440 | 0.001 | 5.83 | 31 | 27 | 5 |
|  | Thalamus | R | 247 | 0.001 | 5.58 | 20 | -29 | 0 |
|  | Posterior-Medial Frontal Gyrus | L | 68 | 0.005 | 5.27 | -9 | -6 | 68 |
| **nAN vs. Baseline** | Fusiform Gyrus  Cerebellum | R | 63088 | 0.001 | Inf | 27 | -78 | 18 |
|  |  |  |  |  | Inf | 36 | -52 | -16 |
|  |  |  |  |  | Inf | 21 | -77 | -11 |
|  | Posterior-Medial Frontal Gyrus  Middle Cingulate Cortex | R/L | 8707 | 0.001 | Inf | -5 | 5 | 54 |
|  |  |  |  |  | Inf | 7 | 8 | 51 |
|  |  |  |  |  | 6.8 | 8 | 12 | 44 |
|  | Postcentral Gyrus  Precentral Gyrus | L | 14929 | 0.001 | Inf | -39 | -32 | 50 |
|  |  |  |  |  | Inf | -36 | -21 | 53 |
|  |  |  |  |  | 7.65 | -36 | -18 | 66 |
|  | Inferior Frontal gyrus  Precentral Gyrus | R | 4714 | 0.001 | 6.82 | 44 | 5 | 32 |
|  |  |  |  |  | 6.19 | 44 | 16 | 23 |
|  |  |  |  |  | 5.87 | 36 | -5 | 51 |
|  | Hippocampus | L | 323 | 0.001 | 6.38 | -19 | -31 | -2 |
|  | Thalamus | R | 272 | 0.001 | 6.07 | 21 | -29 | -2 |
|  | Precentral Gyrus  Inferior Frontal gyrus | L | 884 | 0.001 | 5.91 | -58 | 5 | 33 |
|  |  |  |  |  | 5.85 | -41 | 3 | 30 |
|  | Inferior Frontal gyrus | L | 128 | 0.001 | 5.46 | -29 | 27 | 4 |
|  | Anterior Insula | R | 110 | 0.002 | 5.26 | 31 | 26 | 5 |
|  | Inferior Frontal gyrus | R | 104 | 0.002 | 5.22 | 49 | 32 | 14 |

Results are thresholded at p < 0.05 FEW corrected at the cluster level (cluster size estimated with a voxel-level threshold at p < 0.05 FWE). Local maxima are given in MNI standard brain coordinates. Most probable anatomical regions are derived from Anatomy Toolbox 1.7 (Eickhoff et al., 2005) and listed in “Brain structure” column. AP = Art Pain condition; AN = Art Neutral condition; nAP = non-Art Pain condition; nAN = non-Art Neutral condition.

**Supplementary behavioural results – Aesthetic Judgment Task (AJ)**

Behavioural judgments obtained at the AJ task were submitted to repeated-measures ANOVA with Content (Art, Non-Art) and Emotion (Pain, Neutral) as within-subjects factors. Whenever appropriate, post-hoc comparisons were performed using Bonferroni adjustment. One participant was removed from behavioural analysis due to technical problems in the recording of the responses in the behavioural session. Consequently, behavioural analysis was performed on 19 participants.

Results showed a significant main effect of the factor Content (F_(1,18)_=115.002, p < 0.001), indicating the expected higher aesthetic evaluation attributed to Art (3.861, SE 0.131) than Non-Art stimuli (1.648, SE 0.154). Moreover, a significant interaction Content by Emotion (F_(1,18)_=23.05, p < 0.001) was found (Supplementary Figure 3). Bonferroni post-hoc comparisons showed that Art Pain stimuli (4.029, SE 0.169) were rated more artistically beautiful than Art Neutral stimuli (3.693, SE 0.167; p = 0.021), whereas non-Art Pain stimuli (1.477, SE 0.151) were rated less artistically beautiful than non-Art Neutral stimuli (1.819, SE 0.195; p = 0.018).

Besides the obvious higher aesthetic rating attributed to artistic stimuli than to non-artistic ones, behavioural results showed an interesting interaction which deserves to be briefly discussed, because it demonstrates how the experience of pain changes through the use of art. It has been pointed out that also ugliness can be appreciated and considered as part of aesthetics, so much that we talk about the “aesthetics of ugliness” (Kuplen, 2013; Rosenkranz, 2015). Indeed, artworks may evoke negative aesthetic feelings due to the ugliness depicted, while evoking at the same time pleasure, due to our evaluation of the creative artistic representation of depicted ugliness, as suggested by Kuplen (2013). Moreover, recent neuroimaging studies (e.g., Kawabata and Zeki, 2004; Ishizu and Zeki, 2011; Martín-Loeches et al., 2014) have revealed that beauty and ugliness are not independent aesthetic categories, as they activate the same brain regions, such as the anterior cingulate or the parietal cortex. The recent perspective of “liberated embodied simulation” (Gallese, 2018) - according to which the suspension of the active grip on our daily life during the contemplation of artistic content liberates new simulative energies enhancing the sense of safe intimacy with the fictious world of art - justifies how it is possible in art to appreciate even terrifying contents that we would never appreciate in real life. The present significant interaction between artistic content and emotion fits into this context, corroborating the notion that art allows us to approach and appreciate even contents with a negative value.

**
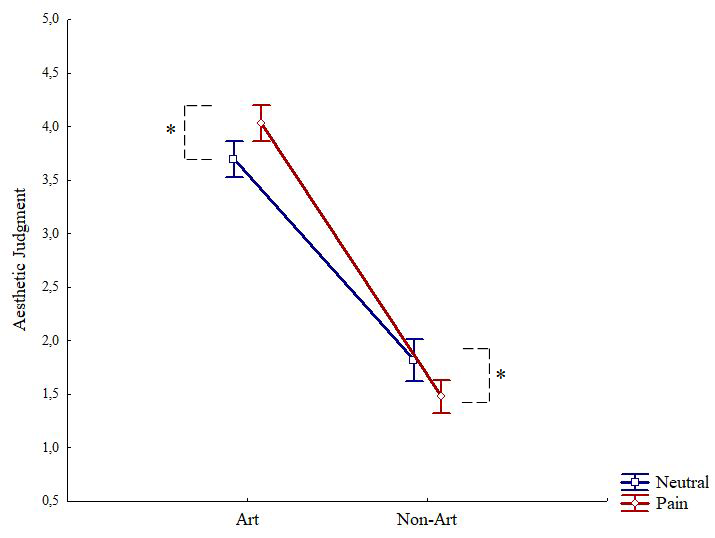
**

**Supplementary Fig. 3. Aesthetic ratings of artistic and non-artistic stimuli. Error bars depicted SE; * = significant differences (p < 0.05) justifying the interaction effect.**
